# Health effects of radioactive contaminated dust in the aftermath of potential nuclear accident in Ukraine

**DOI:** 10.3389/fpubh.2022.959668

**Published:** 2022-08-22

**Authors:** Arash Sharifi, Roshan Dinparastisaleh, Naresh Kumar, Mehdi Mirsaeidi

**Affiliations:** ^1^Department of Marine Geosciences, Rosenstiel School of Marine and Atmospheric Science, University of Miami, Miami, FL, United States; ^2^Isobar Science-Beta Analytic, Research and Development Department, Miami, FL, United States; ^3^Division of Pulmonary and Critical Care Medicine, Johns Hopkins University, Baltimore, MD, United States; ^4^Department of Public Health Sciences, University of Miami, Miami, FL, United States; ^5^Division of Pulmonary and Critical Care Medicine, College of Medicine-Jacksonville, University of Florida, Jacksonville, FL, United States

**Keywords:** health effects, radioactive dust, Ukraine, nuclear accident, HYSPLIT

## One-sentence summary

Our simulation shows widespread radioactive contamination in the event of nuclear accident(s) during the Ukraine war, but the extent and direction of the impact will depend on the timing of the accident.

## Introduction

Thirty-six years have passed since the largest uncontrolled radioactive release at the Chernobyl nuclear power plant in Ukraine. Large amounts of iodine (I) and cesium (Cs) radioisotopes were scattered over a wide area. The total radioactive dose from Chernobyl is estimated at 80,000 man-sieverts, and the death toll was more than 200,000 (1). The current Ukraine war raises serious intentional or unintentional risk of another nuclear disaster. Thus, this research examines the spatiotemporal distribution of potential radioactive dust in the event of accidents in power plants in Ukraine. Here, we modeled air mass movements over the region using the HYSPLIT model and archived meteorological data from April 2021 to March 2022 under the assumption of explosion and release of radioactive particles after referring to as “incident” in any or all Ukrainian nuclear power plants at the same time. Furthermore, we used model outputs to study which countries are likely to be affected by the radioactive dust 3 days after the incident. The health effects of radioactive dust exposure are also discussed based on data collected after the Chernobyl incident.

## Materials and methods

### Site locations

**Figure 1:**
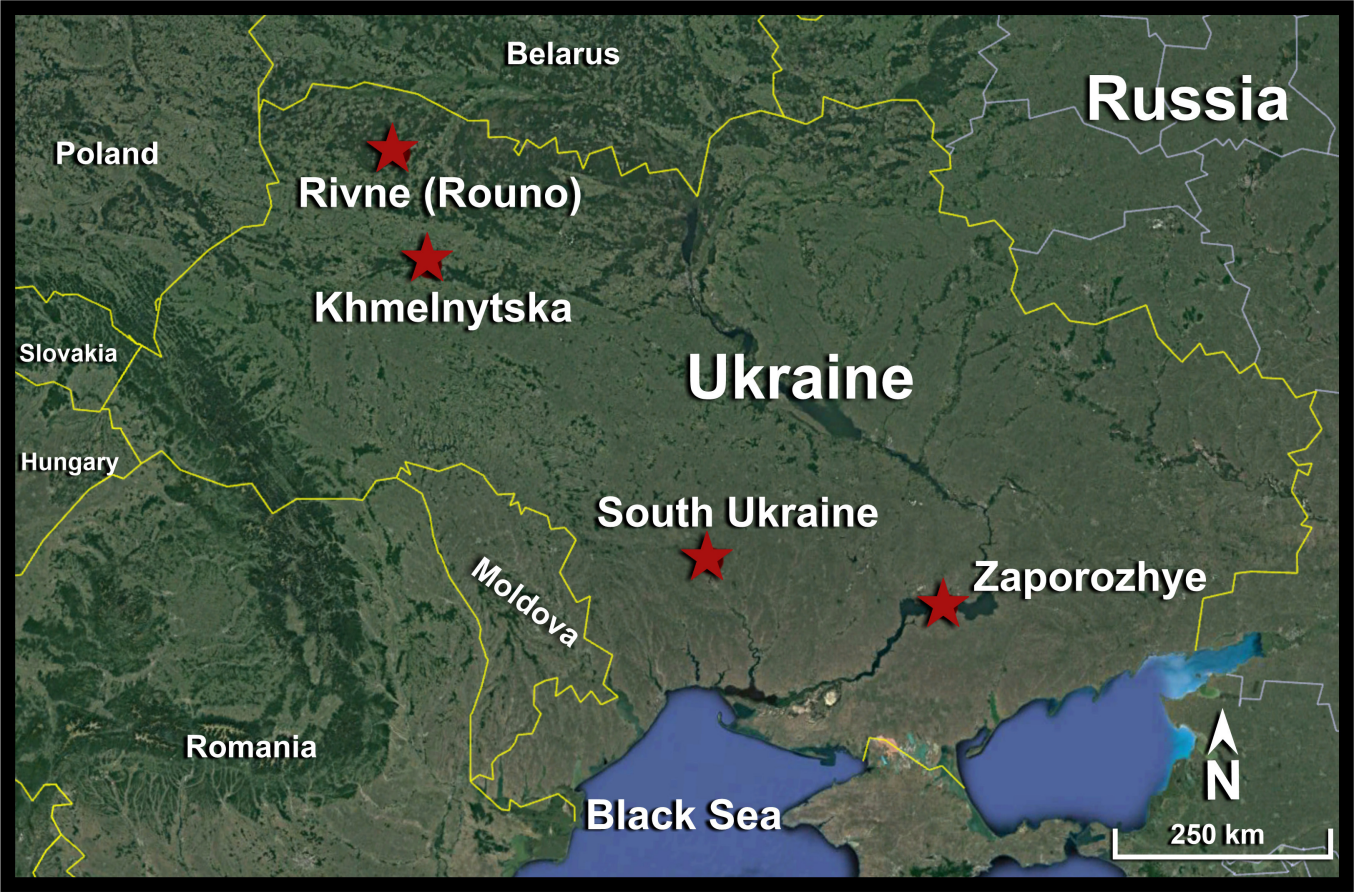
Locations of the Ukrainian nuclear power plants. Base map is from Google Earth.

Ukraine has four operational nuclear power plants with a capacity ranging from 2,000 to 6,000 megawatts and located in the northwest and south of the country [Figure 1 and Table 1 from Global Power Plant Database (2)]. The Rivne (Rouno) and Khmelnytska power plants are located in the northwest almost at the same longitude but 130 km apart, while the South Ukraine and Zaporozhye power plants are located at the same latitude but 255 km away from each other.

**Table 1:** Name, capacity, and coordinates of the Ukrainian nuclear power plants.

**Name**	**Capacity (MW)**	**Latitude**	**Longitude**
Khmelnytska	2,000	50.3024	26.6473
Rivne (Rouno)	2,835	51.3245	25.8974
South Ukraine	3,000	47.812	31.22
Zaporozhye	6,000	47.5119	34.5863

### HYSPLIT forward-trajectory climate simulations

Three-days air mass forward trajectory ensemble plots at 500 m starting altitudes were computed for the first week of each month from April 2021 to March 2022 using the online web version of the HYSPLIT (Hybrid Single-Particle Lagrangian Integrated Trajectory) model (3, 4). HYSPLIT is one of the most extensively used atmospheric transport and dispersion models on earth by the atmospheric sciences community, which has evolved for more than 30 years. Its calculation method is a hybrid between the Lagrangian approach and the Eulerian methodology. HYSPLIT proved to be a complete system for computing not only a simple air parcel trajectory but also complex transport, dispersion, and deposition simulations, and it was successfully used to model the fallout from nuclear clouds (5).

Similarity between trajectories for different timeframes was tested for one of the cites (Rivne) for the first week of each month from April 2020 to March 2021 and from April 2021 to March 2022. The dispersion pattern and movement of air parcel originated from the Rivne site during the aforementioned time frames shows similar pattern (Supplementary Figure S1). Therefore, the time window of April 2021-March 2022 was chosen to represent the atmospheric circulation during the recent 12 months. The starting altitude range for the HYSPLIT model was chosen to be 500–2,000 m to provide maximum coverage for aerosol-bearing air masses that pass over the study area. To incorporate diverse instrumental data into a gridded, 3-D model space, the Global Data Assimilation System with one degree resolution (GDAS1) (https://ready.arl.noaa.gov/archives.php) was used to provide a comprehensive meteorological dataset for the HYSPLIT model. The coordinates of each nuclear power plant were used as the emission source of air masses.

## Result and discussion

**Figure 2:**
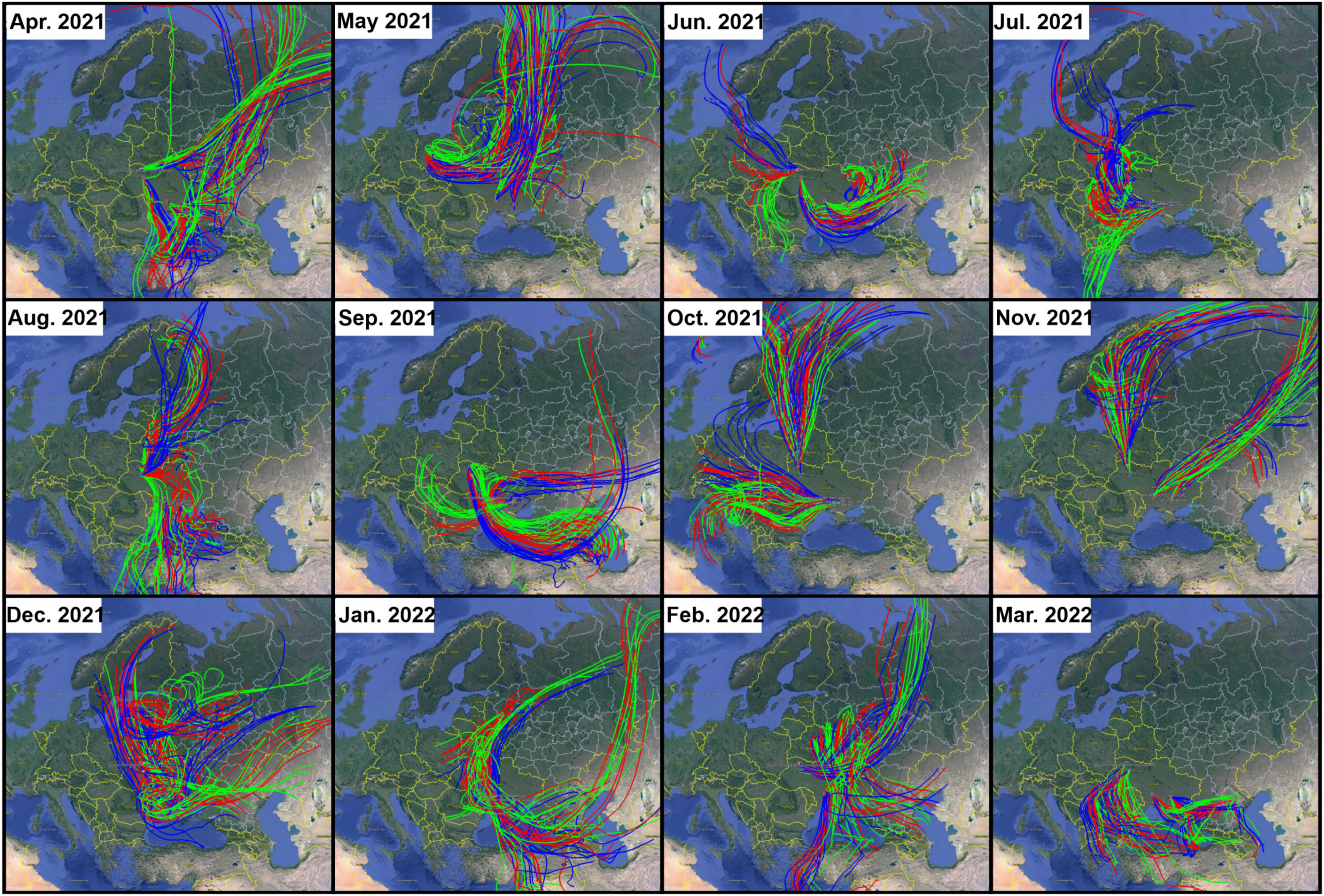
Three-day forward trajectory for the studied sites for the first week of each month starting from April 2021 to March 2022. Base map is from Google Earth.

Results of the HYSPLIT three-days air mass forward-trajectory originated from the locations of the nuclear power plants in Ukraine are shown in Figure 2. As illustrated in Figure 2, the patterns of the air mass movement over the region show a large spatial and temporal variability. This suggests that the radioactive dust generated from the point sources will affect Ukraine, west of Russia, and other countries depending on the timing of the incident during the year (or season). Table 2 summarizes the countries that will be impacted by contaminated air masses during different months of the year. The HYSPLIT trajectories suggest that during the winter months 3 days after the occurrence of an incident in Ukrainian nuclear power plants, countries in central, south, and southeastern Europe as well as countries in the Middle East may receive radioactive-contaminated aerosol. During the fall, the air parcels containing radioactive-contaminated aerosols may hover over the countries in central, north, south, and southeastern Europe. In summer months, air parcels with radioactive-contaminated aerosols may travel over countries in central, north, south, and southeastern Europe, part of the Middle East, north Asia, and even North Africa. Finally, countries in central, north, south, and southeastern Europe may receive contaminated air parcels if the incident happens in Ukrainian nuclear power plants during spring. Major water bodies that could be affected by contaminated air parcels are the Black Sea, Baltic Sea, Mediterranean Sea, and, to a lesser extent, Caspian Sea. The contaminated dust would not reach the United States, at least after the first few days of the potential incident.

**Table 2:** List of countries which contaminated air mass will potentially travel over their air space from April 2021 to March 2022.

**Month**	**Potentially affected countries**
April 2021	Russia, Belarus, Moldova, Romania, Turkey, Cyprus
May 2021	Russia, Belarus, Poland, Lithuania, Latvia, Estonia, Finland
June 2021	Russia, Belarus, Poland, Denmark, Sweden, Norway, Germany, Slovakia, Romania, Moldova, Hungary, Serbia, Kosovo, Macedonia, Greece
July 2021	Russia, Belarus, Poland, Denmark, Sweden, Norway, Germany, Slovakia, Romania, Moldova, Hungary, Serbia, Macedonia, Greece, Libya
August 2021	Russia, Belarus, Lithuania, Latvia, Moldova, Finland, Turkey, Greece
September 2021	Russia, Moldova, Romania, Slovakia, Hungary, Serbia, Czech Republic, Poland, Georgia, Azerbaijan, Kazakhstan, Iran, Turkmenistan
October 2021	Belarus, Lithuania, Latvia, Estonia, Finland, Sweden, Norway, Slovakia, Romania, Serbia, Czech Republic, Germany, Italy, Austria, Switzerland, Croatia, Bosnia and Herzegovina
November 2021	Russia, Belarus, Lithuania, Latvia, Estonia, Finland, Sweden
December 2021	Russia, Belarus, Lithuania, Latvia, Estonia, Poland, Finland, Sweden, Moldova, Romania, Poland, Kazakhstan
January 2022	Russia, Belarus, Lithuania, Latvia, Georgia, Azerbaijan, Turkmenistan, Uzbekistan, Kazakhstan, Turkey, Syria, Iraq, Iran, Lebanon
February 2022	Russia, Belarus, Kazakhstan, Georgia, Turkey, Israel, Syria, Iraq, Lebanon, Jordan
March 2022	Russia, Moldova, Romania, Hungary, Serbia, Kosovo, Macedonia, Bulgaria, Turkey, Greece

The health effects of radioactive dust may be classified as early and late health effects. Acute radiation syndrome (ARS) may occur in the 30 km^2^ around each burst plant resulting in death mainly due to significant skin injuries and bone marrow failure. People who survive from ARS may suffer from cataract, skin injuries, respiratory illnesses such as emphysema and bronchitis, and sexual dysfunction (6–8). Data show that 134 of 600 workers were diagnosed with ARS within hours after the Chernobyl accident. The sickness was seen among the power plant employees and first responders but not in the evacuated population or the general population. Nearly all people with a whole-body dose of more than 6.5 gray (Gy) died within 3 months (9).

Late health effects mainly occur because of development of malignant lesions. A marked increase in thyroid cancer incidence was reported in Belarus, Ukraine, and the four most contaminated regions of Russia (Bryansk, Kaluga, Orel, Tula); among those were younger than 18 years at the time of the Chernobyl nuclear accident. The total number of cases of thyroid cancer due the Chernobyl accident registered between 1991 to 2015 among 18 years or younger in Belarus, Ukraine, and the most contaminated regions of Russia exceeded 19,000 (10). Children and adolescents received high radiation doses to the thyroid mainly because of consumption of fresh milk containing ^131^I, specifically in the 8 weeks following the disaster, and because of iodine's 8-day half-life (11). Individual thyroid doses due to radioactive iodine ingestion fluctuated up to 42 gray (Gy) and depended on the age of the individual, region of exposure, and individual's dairy product consumption habits. Population-average thyroid doses among children of youngest age reached up to 0.75 Gy in Belarus most contaminated area, the Gomel Oblast (12–15).

A Chernobyl cohort of registered recovery and cleanup workers who received radioactive doses varying from 20 to 500 mSv and were followed up over the period 1992–2009 showed a significant increase of 18% in all solid cancers. Furthermore, the dose-response relationship was confirmed (16). In contrast to the substantial increase in thyroid cancer incidence among those exposed at under 18 y, except in clean-up workers, no meaningful evidence of any statistical association between radionuclide exposure and leukemia risk yet exists. Previous studies couldn't establish a consistent correlation between radiation and leukemia incidences in the exposed populations (9, 17, 18).

Nonmalignant diseases may develop in victims of remote exposure to radionuclide dust particles. Since the release of the WHO 2006 report, there has been emerging evidence that radiation as low as 20 mSv can lead to radiation-induced cataracts (19–21) and potential cardiovascular disease (22, 23).

According to experts, psychosocial impacts are the disaster's main public health issues that have affected the largest number of people. Common problems include cerebrovascular diseases, organic mental and mood disorders, and cognitive impairments such as dementia that increased with irradiation dose exposure. Interestingly, radiation exposure level was found to be a common risk factor for mental health effects of the Chernobyl, Fukushima, and 3-mile island nuclear disasters (9, 24, 25).

The current trajectory models are based on the archived data and only present the movement of air masses for the first week of each month. To better understand the movement of air masses that could originate from the Ukrainian power plants and the possibility of countries receiving contaminated air parcels, we recommend a weekly trajectory frequency for each month of the year to be utilized.

## Author contributions

Conceptualization: AS and MM. Methodology and visualization: AS. Investigation and writing-original draft: AS, RD, and MM. Supervision: MM. Writing-review and editing: AS, RD, NK, and MM. All authors contributed to the article and approved the submitted version.

## Conflict of interest

The authors declare that the research was conducted in the absence of any commercial or financial relationships that could be construed as a potential conflict of interest.

## Publisher's note

All claims expressed in this article are solely those of the authors and do not necessarily represent those of their affiliated organizations, or those of the publisher, the editors and the reviewers. Any product that may be evaluated in this article, or claim that may be made by its manufacturer, is not guaranteed or endorsed by the publisher.
