# Supplementary material for: Health effects of radioactive contaminated dust in the aftermath of potential nuclear accident in Ukraine
Source: Front Public Health. 2022 Aug 22;10:959668. doi: 10.3389/fpubh.2022.959668 (PMC9441805; doi:10.3389/fpubh.2022.959668)
Supplement: Supplementary file 1 [file Image_1.pdf]

Supplementary Material

Health effects of Radioactive Contaminated dust in the Aftermath of Potential Nuclear Accident in Ukraine

Authors: Arash Sharifi , Roshan Dinparastisaleh, Naresh Kumar, and Mehdi Mirsaeidi

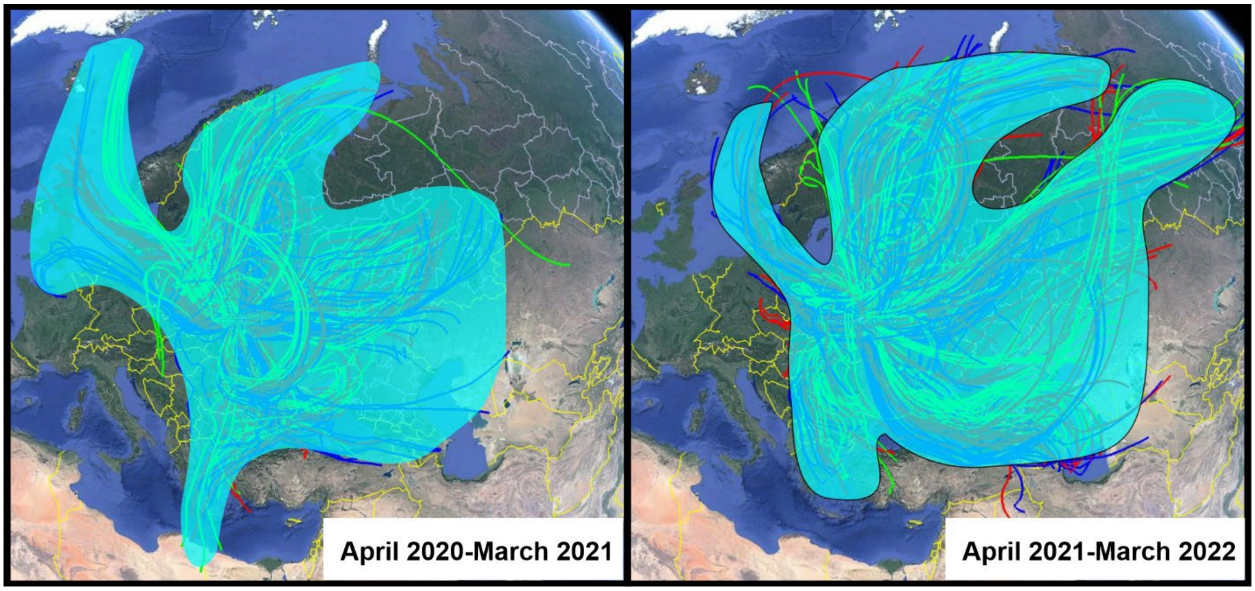

Fig. S-1. Three-days forward trajectory comparison for two time periods, April 2020-March 2021 and April 2021-March 2022 at Rivne nuclear power plant for the first week of each month. Base map is from Google Earth.
